# The associations between mixed blood heavy metal exposure and depressive symptom: a cross-sectional study in Shandong, China

**DOI:** 10.1186/s12889-025-23522-5

**Published:** 2025-07-07

**Authors:** Jing Hou, Yi He, Haifeng Lian, Haosen Yan, Jialian Li, Lailai Yan, Wenzhong Huang, Yiwen Zhang, Sen Gao, Hongwei Sun, Peng Lu

**Affiliations:** 1 School of Public Health, Binzhou Medical University, 346 Guanhai Road, Yantai, 264003 Shandong China; 2 Shanghai Municipal Center for Disease Control and Prevention, Shanghai, China; 3 Department of Service and Management, School of Public Service, Yantai Preschool Education College, Yantai, Shandong China; 4 Peking University, Beijing, China; 5 Department of Epidemiology and Preventive Medicine, School of Public Health and Preventive Medicine, Monash University, Melbourne, Victoria Australia

**Keywords:** Blood heavy metals, Mixtures, Depressive symptoms, Young adults, Environmental risk scores

## Abstract

**Background:**

Given the limited research on the effects of heavy metals on depressive symptoms in young adults and the high prevalence of depression within this age group, it is essential to investigate the potential impact of heavy metals on depressive symptoms.

**Methods:**

This study involved 2027 college students from Shandong, China. Blood concentrations of heavy metals were measured, and depressive symptoms were assessed using the Patient Health Questionnaire-9 (PHQ-9). The Environmental Risk Score (ERS) was used to analyze the relationship between mixed blood heavy metal exposure and depressive symptoms. Sensitivity analyses were conducted using Bayesian Kernel Machine Regression (BKMR) and quantile g-computation (qgcomp) to explore the mixing effect of heavy metals.

**Results:**

A significant positive association was found between ERS and the risk of depressive symptoms. The main effects of silver (Ag), antimony (Sb), tin (Sn), lanthanum (La), and cerium (Ce) were also positively associated with depressive symptoms. Notably, Ce showed an inverted “U”-shaped nonlinear relationship with depressive symptoms risk. La and Ce exhibited antagonistic effects on the increased risk.

**Conclusion:**

In summary, mixed exposure to these five metals may be on the relationship between heavy metal exposure and depressive symptoms in young adults.

**Supplementary Information:**

The online version contains supplementary material available at 10.1186/s12889-025-23522-5.

## Background

Depression is a common mental health condition that significantly adds to the global disease burden, impacting around 300 million individuals across the globe [1]. Recently, depression has emerged as the second leading cause of death among individuals aged 15 to 29 [2], and its prevalence among young people has increased significantly over the past decade [3]. Since young adulthood represents a crucial developmental period [4], people in this age group are more prone to encountering intense psychological distress and face a greater risk of suicide [4]. Consequently, expanding potential interventions to prevent adverse outcomes is essential [5]. Several risk factors for depression have been identified, including stress, behavioral patterns, and sociodemographic factors [6]. Increasing evidence suggests a correlation between heavy metal exposure and mental disorders, specifically depression [7, 8]. Heavy metals contribute to depression-related neurotoxicity by inducing oxidative stress [8–11] which further causes neuroinflammation [10, 12], neurotransmitter imbalance [9, 13], and damage to neural structure and function [14–16], leading to depression-related neurotoxicity, which in turn contributes to the development of depressive symptoms.

Previous studies on the U.S. population have identified an association between exposure to Sn [7] and Sb [8] and depressive symptoms. However, most studies on the Chinese population have focused on the relationship between heavy metal exposure and depressive symptoms in middle-aged and elderly individuals [17, 18]. Fewer studies have investigated the impact of heavy metal exposure on depressive symptoms among young adults. However, young adult populations are often exposed to low levels of metals over long periods, such as through daily exposure to cell phones, computers [19, 20], food packaging [21–23], some crop products [24], and daily toiletries [25]. Therefore, it is particularly important to investigate the link between metal exposure and depressive symptoms in young adult populations.

In the course of their everyday existence, individuals are often subjected to simultaneous contact with a variety of heavy metals. While low doses of chemicals may not produce significant independent effects, their mixed exposure can lead to detectable biological responses [26]. In previous studies, only single metal or mixed metal exposures are usually considered and the complex exposure profiles of mixtures are not taken into account, as the complex exposure profiles of contaminant mixtures may lead to additive, synergistic, or antagonistic effects that cannot be recognized by single contaminant approaches [27, 28]. Experimental studies have also revealed significant synergistic toxicity of multiple metals [29, 30]. Therefore, analyzing mixed exposure to heavy metals and complex exposure scenarios is crucial for a comprehensive understanding of their cumulative toxicity [31].

Given that most studies exploring the association between heavy metals and depressive symptoms have focused on single metals, and that research targeting young populations—who have a high prevalence of depressive symptoms—remains scarce, this study specifically addresses this demographic. We employ the Environmental Risk Score (ERS) method to comprehensively assess the complex relationships between mixed blood heavy metal exposures and depressive symptoms. This approach takes into account main effects, nonlinear relationships, and interactions, thereby more accurately capturing the characteristics of multiple exposures and potential synergistic effects in real-world settings. Through this research, we aim to provide a comprehensive evaluation of the combined heavy metal burden in blood and its association with depressive symptom risk among young people, offering a more refined perspective for environmental mental health studies.

## Materials and methods

### Study sample

This study recruited participants from the Chinese Undergraduate Cohort (CUC) in September 2019, as previously described in detail [32]. In brief, the CUC enrolled participants from Binzhou Medical University in Yantai, Shandong Province. Basic information, including age, gender, height, weight, family income, smoking, alcohol consumption, and history of diseases, was collected through self-report questionnaires. A total of 2,743 participants completed the questionnaire. Each participant also provided a self-administered questionnaire and a 10 ml fasting blood sample. Blood samples were collected between 7 a.m. and 9 a.m. daily from September 2 to September 10, 2019. After excluding participants without whole blood samples or those who experienced a major emotional shock or took medication recently (*n* = 716), a final of 2,027 participants were recruited in the study (Figure S1). All study participants were required to provide written informed consent, and this study received approval from the ethics committee of Binzhou Medical University.

### Elemental detection

On the scheduled morning, 10 ml of fasting peripheral venous blood was collected from each participant at the university hospital and stored at -80 °C for backup. An inductively coupled plasma-mass spectrometer (ICP-MS, ELAN DRC II, PerkinElmer, USA) was used to measure the levels of the elements. In brief, blood samples (0.35 ml) were transferred into quartz tubes and then mixed with 0.40 ml of nitric acid. We pre-digested the quartz tubes at ambient temperature for two hours and then placed them in an Ultra WAVE microwave digestion system (Ultra WAVE, Milestone Co., Italy) for 50min. Afterward, we added 0.1 ml of internal standard elements (Indium: 2 ng/ml) and diluted to 8 ml with ultrapure water. The analysis was performed in the Central Laboratory of Biological Elements in the Peking University Health Science Center, certified by the Chinese Metrology Accreditation (CMA) system.

### Diagnosis of depressive symptoms

The status of Depressive Symptoms Among Participants was assessed by the Patient Health Questionnaire-9 (PHQ-9), and each of the 9 items was scored on a scale from 0 (not at all) to 3 (nearly every day), yielding a total score that ranges from 0 to 27 [33]. The PHQ-9 is grounded in the criteria for major depressive disorder as articulated in the Fourth Edition of the Diagnostic and Statistical Manual of Mental Disorders (DSM-IV). It has been recognized as both trustworthy and accurate for utilization within the general population [34]. A total score ≥ 10 was used to identify individuals with depressive symptoms, given the high sensitivity (≥ 0.85) and specificity (≥ 0.75) of PHQ-9 at this threshold across diagnostic interview-based validations [35]. The scale has demonstrated strong reliability, with Cronbach’s *α* = 0.874 reported in a large study of Chinese university students (*N* = 12,858) [36], which is consistent with our observed internal consistency (*α* = 0.892). Furthermore, previous clinical validation studies have shown good test-retest reliability (*r* = 0.84) [35].

### Statistical analysis

Spearman pairwise correlations were calculated for 18 metals, resulting in a correlation matrix heat map. The application of a logarithmic transformation with a base of 10 to metals was driven by two key observations. Firstly, the data exhibited a high degree of skewness in the distribution of the raw values. Secondly, the shapes of the dose-response associations were found to be more linearly associated when subjected to the log transformation. The difference between depressed and non-depressed groups was assessed using the Mann-Whitney test for non-normal continuous variables, while categorical variables were analyzed using either the Kruskal-Wallis test or the Chi-square test. A review of the literature revealed the existence of multiple confounding factors, including age [23], sex [24], BMI [25], family income [26], and lifestyle behavior (smoking, alcohol consumption, physical activity) [27], and they were controlled in the multivariate model.

The Adaptive Elasticity Network (AENET) was selected as the main method for screening depression-related heavy metals. AENET is an adaptive version of the elastic net that addresses the collinearity problem present in the elastic net and satisfies the asymptotic normality assumption [27]. We included a total of 18 heavy metals, and 189 predictors could be used for model selection, including 18 main effect terms of heavy metals, 18 quadratic terms, and 153 pairwise linear interaction terms. AENET performs variable selection through 10-fold cross-validation. Finally, a total of 5 variables were selected from 189 candidate predictors. Ag and Sb had two main effects, the quadratic terms Sn and Ce, there is an interaction term between La and Ce.

The ERS was principally utilized for the purpose of evaluating the correlation between exposure to mixed heavy metals and symptoms of depression. This score has proven to be a valuable instrument for evaluating the accumulated risk of disease resulting from contact with chemical mixtures [27, 37]. ERS, as a composite measure of the impacts of multiple pollutants, is capable of simultaneously evaluating the main effects, squared terms, and interaction effects of multiple pollutants [38]. The squared terms captured non-linear connections between metals and depressive symptoms, while the interaction terms indicated possible deviations from purely additive combined effects. The calculation formula [38] is

$$\begin{aligned}{\rm{ER}}{{\rm{S}}_{\rm{i}}} =& \sum\limits_{j = 1}^P {{{\widehat \beta }_j}} Z_i^j + \sum\limits_{k = 1}^{P - 1} {\sum\limits_{l = k + 1}^P {{{\widehat \beta }_{kl}}} } Z_i^kZ_i^l \\&+ \sum\limits_{m = 1}^P {{{\widehat \beta }_m}} {\left( {Z_i^m} \right)^2}\end{aligned}$$

$$\begin{aligned}{\rm{ERS}} = &{\beta _1}Ag + {\beta _2}Sb + {\beta _3}S{n^2} + {\beta _4}C{e^2} \\&+ {\beta _5}\left( {La \times \:Ce} \right)\end{aligned}$$

For the interpretability of the interaction and nonlinear effects, we included the main effects of all predictors screened by AENET together in the logistic model. The value of *β* was derived from a logistic model that incorporated the observed matrix of pollutant concentrations for each participant. This model included the main effects, squared terms, and interaction terms. The objective was to obtain weighted concentrations. The weighted concentrations were summed to calculate individual ERS, which reflected the weighted total of heavy metal mixture exposure. Higher ERS values indicate a higher incidence of depressive symptoms associated with exposure to heavy metal mixtures. Generalized linear regression analysis was performed to explore the effect of ERS on the risk of depressive symptoms. Thereafter, ERS was categorized into quintiles, and multinomial logistic regression was utilized to explore the relationship between increasing quintiles of ERS and the risk of depressive symptoms. The association intensity between exposure to heavy metals and graded depressive symptom severity was quantitatively evaluated using Odds Ratios (OR) in the statistical analysis. Additionally, the Restricted Cubic Spline Regression model (RCS) was utilized to investigate the dose-response correlation regarding heavy metals and depressive symptom risk.

Stratified analyses were performed to identify susceptible populations. Analyses were stratified by sex and BMI (overweight or obesity was defined as BMI ≥ 24 kg/m^2^, normal or thin was defined as BMI < 24 kg/m^2^).

In order to guarantee the reliability of the results obtained, several sensitivity analyses were performed. The reliability of the primary model was systematically evaluated across diverse experimental configurations, excluding covariates or including additional covariates (history of heart disease, hypertension, hyperlipidemia, and diabetes mellitus). We then used BKMR and qgcomp models to assess the mixed effects of metal mixtures on depression symptoms.

Statistical analyses were conducted utilizing R (version 4.3.1), employing the “gcdnet”, “qgcomp”, and “bkmr” packages for the corresponding analyses. The significance level was set as *p*-value < 0.05 (two-sided).

## Results

### Participants’ characteristics

Table 1 shows the demographic characteristics of the 2027 participants. Our analysis revealed increased rates of depression among individuals falling into two income categories: ≤ 7000USD and 21,000 ~ 28000USD. In addition, smokers, alcohol consumers, and individuals with no physical activity also showed higher rates of depressive symptoms. In Table S1, we present the median levels of 18 blood heavy metals for all participants, as well as separate data for males and females. Males had higher concentrations of iron, zinc, germanium, and mercury compared to females. Conversely, silver, chromium, copper, manganese, lanthanum, cobalt, and nickel were lower in men than in women. Spearman’s correlation analysis demonstrated varying degrees of association (ranging from 0.1 to 0.7) among most heavy metals, as depicted in Figure S2.

**Table 1:** Demographic characteristics of the study population (*N* = 2027)

Characteristics	Non-depressed (*N* = 1922, %)	Depressed (*N* = 105, %)	*P*
**Age (years)**	18.39 ± 0.64	18.30 ± 0.65	0.31
**Sex**			
Female	1117(94.82)	61(5.18)	0.99
Male	805(94.82)	44(5.18)	
**BMI (kg/m**^**2**^)	22.45 ± 5.00	22.42 ± 4.35	0.68
**Family income (USD)**			
≤ 7000	773(92.91)	59(7.09)	< 0.01
7000 ~ 14,000	700(96.29)	27(3.71)	
14,000 ~ 21,000	281(97.91)	6(2.09)	
21,000 ~ 28,000	85(90.43)	9(9.57)	
≥ 28,000	83(95.4)	4(4.60)	
**Smoking**			
No	1902(94.96)	101(5.04)	0.03
Yes	20(83.33)	4(16.67)	
**Alcohol consumption**			
No	1827(95.06)	95(4.94)	0.04
Yes	95(90.48)	10(9.52)	
**Physical activity**			
No	423(91.96)	37(8.04)	< 0.01
Yes	1499(95.66)	68(4.34)	

### Selection of heavy metals and ERS construction

In the AENET model, a total of five variables were selected from 189 candidate predictors. Two main effects, Ag, Sb; two quadratic terms, Sn, Ce; and one interaction term between La and Ce. We also included the main effects of Sn, La, and Ce above fitting a logistic model in order to make the interaction terms and nonlinear effects interpretable (Table 2). For the ERS construction, the *β* was derived from a logistic model that incorporated the observed matrix of pollutant concentrations for each participant. This model included the main effects, squared terms, and interaction terms. The objective was to obtain weighted concentrations. The weighted concentrations were summed to calculate individual ERS, which reflected the weighted total of heavy metal mixture exposure. Higher ERS values indicate a higher incidence of depressive symptoms associated with exposure to heavy metal mixtures.

**Table 2:** Comparison of *β* values of AENET and logistic model

	AENET	Logistic
**Main effects**		
Ag	0.102	0.174
Sb	0.051	0.128
Sn	-	0.149
La	-	0.026
Ce	-	0.058
**Square terms**		
Sn	-0.089	-0.173
Ce	-0.090	-0.065
**Pairwise interactions**		
La&Ce	-0.131	-0.437

### Relationship between ERS and depressive symptoms

**Fig. 1:**
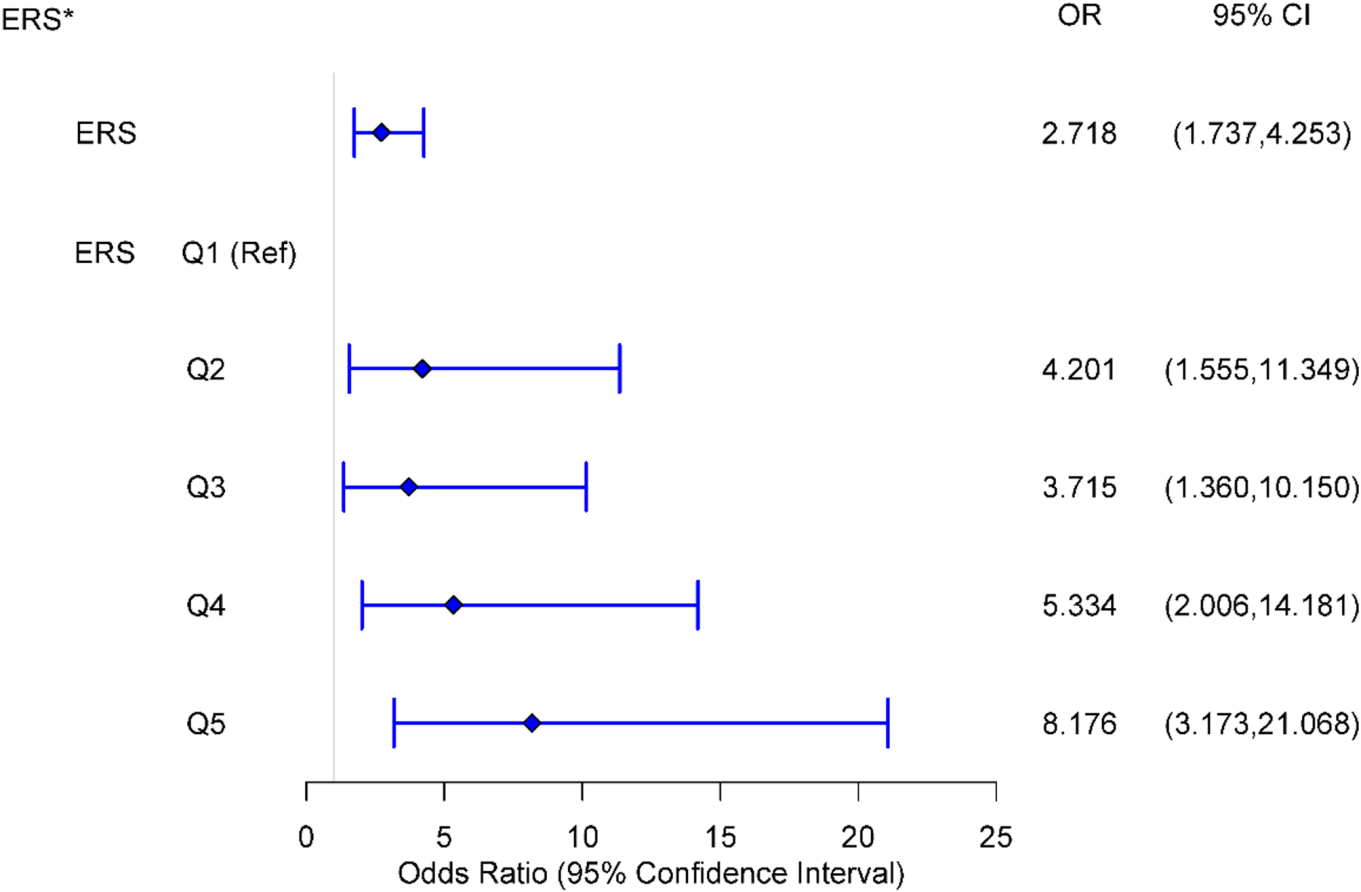
The probability of depressive symptoms after ERS quintile transformations (reference Q1). *: The logistic model was adjusted for age, sex, BMI, smoking, alcohol consumption, physical activity, and family income. The adjusted ORs showed that Q2, Q3, Q4, and Q5 of ERS were positively associated with depressive symptoms compared to Q1

We created an ERS using the identified heavy metals. The findings of the model are indicative of a substantial positive connection between ERS and depressive symptoms. Each incremental unit elevation in the ERS was significantly correlated with a 2.718-fold augmentation in the probability of depression onset (OR: 2.718; 95% CI: 1.737 ~ 4.253). Figure 1 shows the results of transformed dummy variables for ERS quintiles included in the logistic model. The adjusted ORs showed that depressive symptoms were found to be positively related to Q2, Q3, Q4, and Q5 of ERS in comparison to Q1. The corresponding OR and 95% CI were 4.201 (95% CI: 1.555 ~11.349); 3.715 (95% CI: 1.360 ~10.150); 5.334 (95% CI: 2.006 ~14.181); 8.176 (95% CI: 3.173 ~21.068).

### Association of individual heavy metals with depressive symptoms

**Fig. 2:**
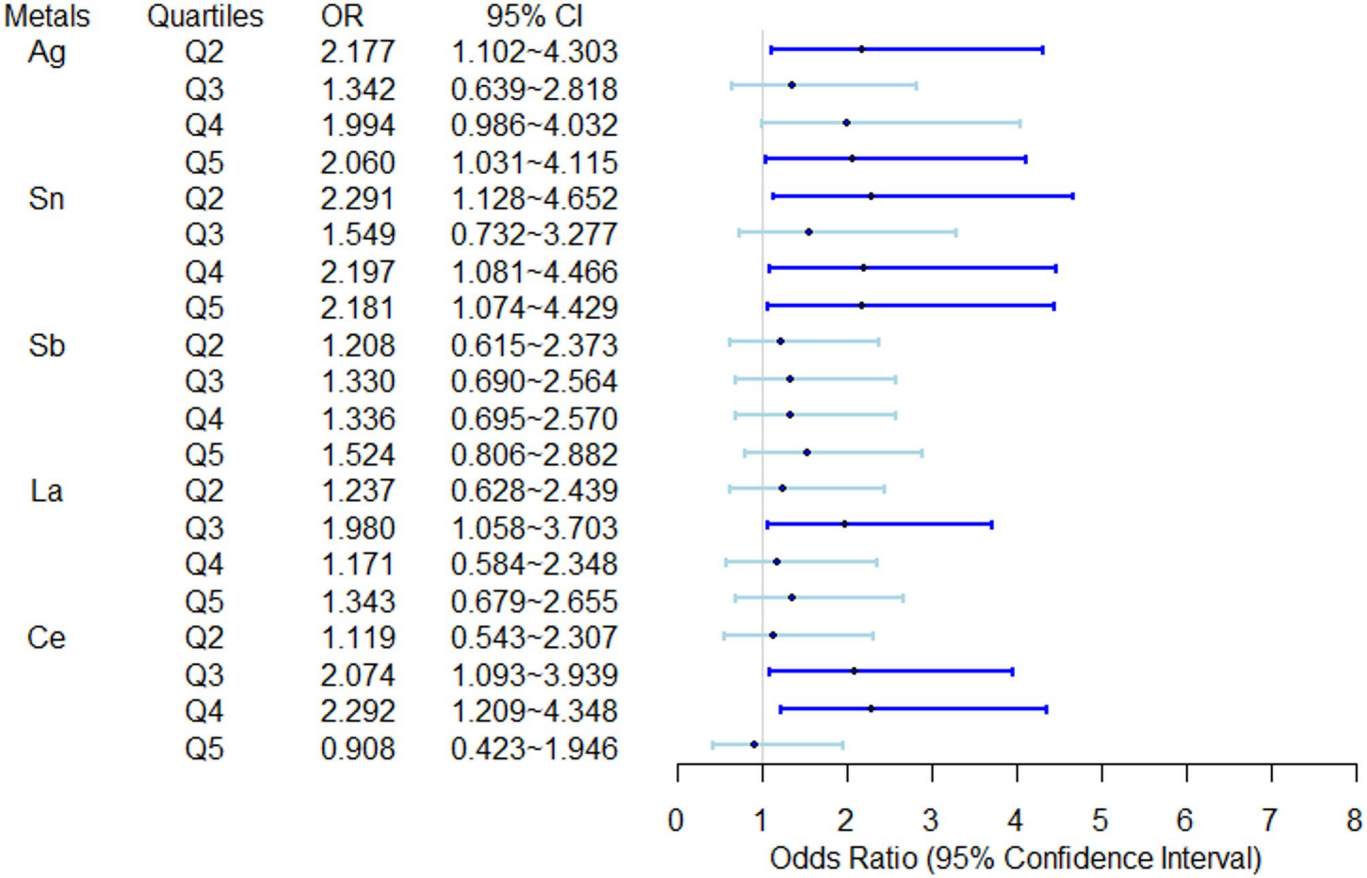
Independent (compared to the first quintile) effects of heavy metals on depressive symptoms. The logistic model was adjusted for age, sex, BMI, smoking, alcohol consumption, physical activity, and family income. Q2, second quintile; Q3, third quintile; Q4, fourth quintile; Q5, fifth quintile; Dots, ORs; lines, 95% confidence interval; dark blue denoted significant associations, *P* value < 0.05. When compared to the first quintile, individuals exposed to Ag at the second quintile level, Sn at the second, fourth, and fifth quintile levels, La at the third quintile level, and Ce at the third and fourth quintile levels exhibited higher odds of experiencing depressive symptoms

In the logistic model, a positive correlation was demonstrated between the quintile exposure levels of heavy metals (Ag, Sn, La, Ce) and depressive symptoms, as depicted in Fig. 2. When compared to the first quintile, individuals exposed to Ag at the second quintile level, Sn at the second, fourth, and fifth quintile levels, La at the third quintile level, and Ce at the third and fourth quintile levels exhibited higher odds of experiencing depressive symptoms. Table S2 provides the association results between all 18 heavy metals and depressive symptoms. Figure S3 depicts the dose-response correlation between five heavy metals and depressive symptoms through restricted cubic spline regression models.

### BKMR and qgcomp models to assess mixed heavy metal exposure

**Fig. 3:**
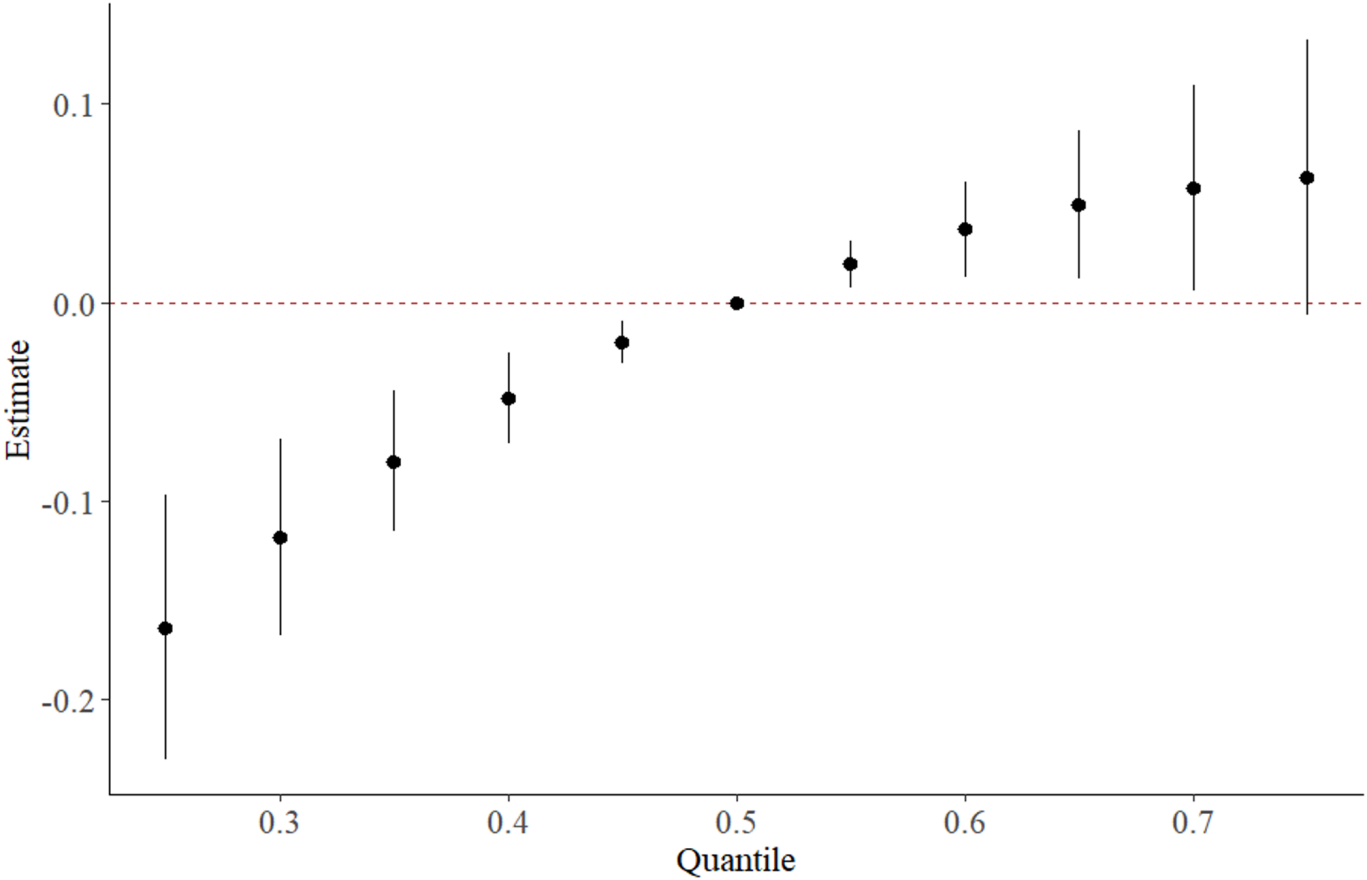
Exploring the effects of mixed exposure to heavy metals Ag, Sb, Sn, La, Ce on depressive symptoms using the BKMR model

Figure 3 depicts the estimates of the risk of depressive symptoms with percentile changes of heavy metal mixture exposure from the BKMR model. The findings reveal a linear increase in depression risk with a rising percentile of metal mixture concentration. Figure S4 presents the dose-response correlation between a metal’s exposure level and depressive symptoms when other metals are at median concentrations. Sn and Ce show a nonlinear correlation. The risk of experiencing depressive symptoms rose and then fell as the concentration of heavy metals increased. Furthermore, Figure S5 demonstrates an antagonistic effect between La and Ce, confirming the results obtained in AENET. The qgcomp mixture exposure results showed a statistically significant heavy metal mixture effect with depressive symptoms (OR: 1.361; 95% CI: 1.106 ~ 1.615) (Table S3) (Figure S6).

### Stratified and sensitivity analysis

In the logistic model, a positive correlation between ERS and depressive symptoms was detected within specific subgroups (Figure S7). Specifically, ERS was discovered to have a positive association with depressive symptoms in both males and females (OR: 3.390; 95% CI: 1.578 ~ 7.279, OR: 2.415; 95% CI: 1.384 ~ 4.214). The difference between the odds ratios for men and women was not statistically significant (*P* = 0.482). Additionally, a significant association was observed between ERS and depressive symptoms in overweight or obese individuals (BMI ≥ 24 kg/m^2^) (OR: 3.522; 95% CI: 1.329 ~ 9.337), as well as in individuals of normal weight or lean (BMI < 24 kg/m^2^) (OR: 2.557; 95% CI: 1.537 ~ 4.253). The discrepancy between these two odds ratios did not attain a statistical significance level (*P* = 0.568).

In order to verify the stability of the main model, we debugged the main model by decreasing and increasing the adjustment covariates. These results are essentially identical to those of the main model, suggesting that the main findings are consistent and dependable (Figure S8).

## Discussion

To the best of our understanding, this is the initial study to evaluate the association between cumulative exposure to a combination of heavy metals and depressive symptoms in young adults. In addition to assessing the main effects of individual metals, we included nonlinear effects and interaction effects, which better reflect real-world scenarios of heavy metal mixture exposure. An ERS was constructed in order to capture the impact of the accumulation of metal exposure on the manifestation of depressive symptoms in young adults. The study identified the adverse effects of the major metals Ag, Sb, Sn, La, and Ce on depressive symptoms. The present findings offer a novel perspective on the effects of combined heavy metal exposure on mental health.

In the present study, the incidence of depressive symptoms among the subjects was ascertained to be 5.18%. According to the first nationwide survey of mental disorders in China, this rate exceeded that observed in young adults (3.6%) [39]. The disparity in prevalence may be attributed to our study population consisting of university students, who have been shown to exhibit a higher incidence of depressive symptoms in comparison to the population as a whole or non-university students [40]. However, our finding of depressive symptoms prevalence was lower than the 9.7% reported by Hou et al. [41]. This variation could be attributed to using the PHQ-9 scale for identifying depressive symptoms in our study, whereas theirs depended on the Symptom Checklist-90 (SCL-90) [41].

The present study established a significant association between exposure to a mixture of heavy metals (Ag, Sb, Sn, La, and Ce) and an elevated risk of depressive symptoms. This indicates that young individuals with higher exposure to these metals are more likely to develop depressive symptoms. These findings align with previous population-based studies. Fu et al. [42] reported that exposure to individual metals not only had adverse health effects but also amplified the probability of developing depression when combined with exposure to a mixture of metals (Sb, Sn, and Cd). A positive correlation between Sb and depression was also found.

Ag, Sb, Sn, La, and Ce can be encountered through shared exposure pathways, particularly via consumer products and environmental media. Sb and Sn are present in electronics (e.g., flame retardants, solder, and circuit coatings) and may expose students to dust or skin contact during frequent device use [19, 20]. Sb is also used in PET bottles, while Sn in food cans can leach under acidic conditions [21–23]. La and Ce, common in rare earth fertilizers, may enter the body through the food chain [24]. Ag nanoparticles in personal care items such as toothpaste and skin products could be absorbed via skin or mucosa [25]. These shared pathways contribute to potential long-term, low-dose exposure among university students.

Emerging evidence suggests heavy metals influence depressive symptoms through interconnected biological pathways: Oxidative stress induced by Sb and Ag nanoparticles causes hippocampal damage via ROS generation [8–11]. Neuroinflammation triggered by Ce compounds and prenatal Ag exposure disrupts hippocampal signaling [10, 12]. Neurotransmitter dysregulation occurs through Ag-altered serotonin/dopamine levels and Sb-inhibited AChE activity [9, 13]. Structural impairments involve Sn-reduced reelin expression and La-mediated ANLS inhibition in the hippocampus [14–16]. These mechanisms collectively contribute to depression-related neurotoxicity.

Sb has been recognized as a priority pollutant by both the European Union and the United States Environmental Protection Agency due to its potential carcinogenic properties. Additionally, some researchers have classified it as a novel nerve poison [9]. In our study, a positive correlation was identified between Sb and depressive symptoms, suggesting that individuals exposed to elevated Sb levels were at an increased risk of developing depressive symptoms. However, the existing literature on the relationship between Sb and depression remains inconclusive. A study utilizing NHANES data from 2007 to 2016 revealed a noteworthy nonlinear adverse impact of Sb on depression risk [8]. Conversely, Shiue’s research indicated no link association between exposure to Sb and vulnerability to depression and did not investigate the dose-response relationship [7]. Experimental studies have found that mouse and aquatic animal models exposed to Sb for some time showed elevated levels of pro-inflammatory factors and pro-oxidant substances, which were significantly associated with the onset and severity of depression [8, 9]. A study was conducted that demonstrated an inverse correlation between urinary antimony levels and telomere length [43]. In addition, another study indicated that telomere shortening may serve as a precursory phenomenon and potential risk element in the onset of depression [44].

Our research found that Sn in the blood increases the risk of depressive symptoms. The uses of Sn are various, including stabilizers in plastics, antifoam in paints, preservatives in some food cans, and pesticides [22, 23]. Despite the paucity of studies reporting on the toxic effects of inorganic tin, organotin has been determined to be an endocrine disrupter, with tributyltin(TBT) being the specific compound in question [45]. Both Sn and Sb have been implicated in influencing gene expression in zebrafish models exposed to TBT [46, 47]. Consequently, Sn may contribute to an imbalance in oxidative and antioxidative status, as well as disrupt neuroendocrine factors, potentially leading to depression. Sn can cross the blood-brain barrier to produce neurotoxicity [48]. Some experimental results support that exposure to Sn may cause the dysfunction of brain regions associated with emotion regulation (e.g., cerebellum, hippocampus, hypothalamus, striatum) [14, 15, 49, 50]. For example, exposure to Sn decreased the level of reelin protein expression within the hippocampus of rats [14], possibly leading to depression [15].

Our analysis revealed a positive relation between Ag concentrations and the manifestation of depressive symptoms. To our knowledge, limited epidemiological research has been conducted regarding the impact of Ag on depressive symptoms. However, some experimental studies have found that Prolonged contact with Ag particles resulted in depressive responses in rodents [51]. Due to their small size, Ag particles easily cross several biological barriers, affecting brain development [10], causing damage to hippocampal function [11], and affecting neurotransmitter levels such as dopamine and serotonin [13]. Serotonin has long been acknowledged as a pivotal regulator of mood and cognitive function, exhibiting a strong association with the etiology of major depression [52, 53]. Dysfunction of the dopamine system in the brain’s reward circuitry is associated with depression, with depressed patients having lower dopamine concentrations than non-depressed individuals [54].

We discovered that the risk of depressive symptoms increased as Ce levels rose, before decreasing again. La and Ce had antagonistic effects on the increased risk of depressive symptoms. The observed inverted U-shaped association between exposure to Ce and depression risk (*P* = 0.016) may reflect its context-dependent dual roles: under high oxidative stress conditions (e.g., neurodegenerative or depression models), CeO_2_NPs exert neuroprotective effects through antioxidative and anti-inflammatory actions [55, 56] whereas in healthy systems, excessive Ce may disrupt normal redox signaling and exhibit pro-oxidant effects [12]. Karg-Gemici et al. [57] further support this biphasic nature by showing that CeO_2_NP pretreatment alleviates isoflurane-induced neurotoxicity in neonatal rats, while also underscoring the need for dose optimization due to side effects observed in healthy controls. Additionally, other researchers have observed that LaCl_3_ negatively affects spatial learning and memory in rats, with the mechanism likely involving suppression of ANLS(astrocyte-neuron lactate shuttle)in the hippocampus [16]. The antagonistic interaction between Ce and La may arise from toxicokinetic competition for absorption/excretion pathways [58] and shared transport channels due to their similar ionic radii, as shown in algae [59], collectively reducing bioaccumulation and toxicity. These findings imply a potential association between exposure to La and Ce and depression. However, conducting more studies to prove and confirm this relationship is essential.

Our findings demonstrate a higher prevalence of depressive symptoms among smokers and alcohol drinkers, despite their smaller subgroup sizes—a phenomenon consistently observed across multiple studies [60, 61]. This association may reflect shared psychosocial vulnerabilities predisposing individuals to both substance use and depression, as well as potential self-medication behaviors where substances temporarily alleviate depressive symptoms [62]. Although limited by self-reporting bias and the cross-sectional design, our models adjusted for smoking/alcohol consumption status, confirming that heavy metals’ effects on depression persist independently. Future studies should investigate the temporal relationships and biological interactions between metal exposure and substance use in depression etiology.

The present study is notable for several key strengths. First, to the best of our knowledge, this is the initial study to analyze the effects of multiple blood metals on depressive symptoms in young adults, a population with a relatively high prevalence of depression. Unlike previous studies, the present study used the ERS to comprehensively assess the association between mixed exposure to heavy metals and depressive symptoms, taking into account the main, nonlinear, and interaction effects of heavy metals. Second, we collected blood from participants as biological samples for analysis, considering that blood-based internal exposure measurement is more accurate in assessing the true exposure level of the human body compared to external exposure measurement [63]. Meanwhile, there are also some limitations that need to be addressed. First, the cross-sectional nature of the present study precludes the determination of an association between long-term exposure to heavy metals and the persistence of depressive symptoms. Second, the metals utilized in this study exhibit varying half-lives in human subjects, consequently resulting in disparate measurement error degrees. Thirdly, unmeasured psychosocial variables such as academic stress levels, and peer relationship quality may potentially influence depressive symptoms, although their biological association with internal metal concentrations remains weak, resulting in minimal bias from such omissions. Finally, although our study participants were distributed throughout Shandong Province (Figure S8), our participants were undergraduate students at Binzhou Medical College, which may limit the generalization of the findings to all young people.

## Conclusion

In summary, the current study suggests that mixed blood heavy metal exposure may increase the likelihood of depressive symptoms. Specifically, metals linked to such symptoms include Ag, Sb, Sn, La, and Ce. Further, longitudinal studies are warranted to validate the influence of long-term exposure to these heavy metals on depressive symptoms. Considering the prevalence of low levels of metals in the environment and their impact on psychological problems, it is necessary to strengthen the prevention and control of heavy metal pollution and improve the relevant management system. At the same time, when setting the health threshold of heavy metals, environmental monitoring, and exposure assessment should be carried out on university campuses and other places where young people are concentrated. Especially the potential heavy metal contamination in drinking water, canteen food, and daily necessities.

## Electronic supplementary material

Below is the link to the electronic supplementary material.

Supplementary Material 1

## Data Availability

No datasets were generated or analysed during the current study.
