# Supplementary material for: The associations between mixed blood heavy metal exposure and depressive symptom: a cross-sectional study in Shandong, China
Source: BMC Public Health. 2025 Jul 7;25:2405. doi: 10.1186/s12889-025-23522-5 (PMC12235819; doi:10.1186/s12889-025-23522-5)
Supplement: Supplementary file 1 — Supplementary Material 1 [file 12889_2025_23522_MOESM1_ESM.docx]

**Supplementary Material for “The associations between mixed blood heavy metal exposure and depressive symptom: a cross-sectional study in Shandong, China”**

1. **Table S1.** Median level of heavy metals in whole blood of university students.
2. **Table S2.** Single heavy metal model results.
3. **Table S3.** Qgcomp Model: Association between heavy metal mixtures and depressive symptoms.
4. **Figure S1** Sample selection flow chart
5. **Figure S2.** The correlation among heavy metals.
6. **Figure S3.** Cubic spline modeling of five depressive symptom-related metals (Ag, Sb, Sn, La, and Ce).
7. **Figure S4.** BKMR model to assess nonlinear associations of five heavy metal exposure levels with depressive symptoms
8. **Figure S5.** Results of interaction between heavy metals.
9. **Figure S6.** The Qgcomp method assesses the association between mixed exposure to five metals and depressive symptoms.
10. **Figure S7.** Relationship between ERS and depressive symptoms in different subgroups.
11. **Figure S8** Sensitivity analysis of ERS and risk of depression.
12. **Figure S9.** Distribution of study subjects across Shandong Province.
13. **Elemental detection**

**Table S1** Median level of heavy metals in whole blood of university students (ng/ml)

| Element | Our Study (Median (P_25_, P_75_)) | | | *P* for sex difference |
| --- | --- | --- | --- | --- |
|  | Total | Male | Female |  |
| Silver (Ag) | 0.10 (0.07, 0.14) | 0.09 (0.07, 0.13) | 0.10 (0.07, 0.15) | <0.01 |
| Tin (Sn) | 0.60 (0.38, 0.90) | 0.61 (0.37, 0.94) | 0.58 (0.38, 0.88) | 0.31 |
| Stibium (Sb) | 4.76 (4.22, 5.54) | 4.74 (4.17, 5.52) | 4.76 (4.27, 5.57) | 0.16 |
| Neodymium (Nd) | 0.12 (0.09, 0.16) | 0.12 (0.09, 0.15) | 0.12 (0.09, 0.16) | 0.38 |
| Iron (Fe, ×10^5^) | 5.01 (4.36, 5.59) | 5.45 (5.05, 5.92) | 4.60 (4.03, 5.15) | <0.01 |
| Zinc (Zn, ×10^3^) | 5.57 (4.82, 6.30) | 5.91 (5.32, 6.57) | 5.22 (4.58,5.97) | <0.01 |
| Chromium (Cr) | 2.09 (1.13, 3.75) | 1.98 (1.07, 3.45) | 2.15 (1.19, 4.00) | 0.01 |
| Germanium (Ge) | 75.00 (58.89, 90.70) | 80.24 (64.06, 95.32) | 71.32 (55.08, 86.48) | <0.01 |
| Molybdenum (Mo) | 1.46 (1.17, 1.84) | 1.44 (1.16, 1.79) | 1.48 (1.18, 1.88) | 0.06 |
| Cadmium (Cd) | 1.27 (1.04, 1.55) | 1.27 (1.05, 1.52) | 1.28 (1.02, 1.57) | 0.38 |
| Cuprum (Cu, ×10^2^) | 8.53 (7.69, 9.52) | 8.19 (7.41, 9.12) | 8.79 (7.92, 9.87) | <0.01 |
| Mercury (Hg) | 0.73 (0.54, 0.99) | 0.77 (0.56, 1.05) | 0.71 (0.52, 0.94) | <0.01 |
| Manganese (Mn) | 15.66 (12.69, 19.40) | 14.23 (12.01, 17.19) | 16.99 (13.64, 20.88) | <0.01 |
| Lanthanum (La) | 0.04 (0.02, 0.06) | 0.03 (0.02, 0.05) | 0.04 (0.02, 0.06) | 0.03 |
| Cerium (Ce) | 0.11 (0.08, 0.15) | 0.11 (0.08, 0.14) | 0.11 (0.08, 0.15) | 0.68 |
| Praseodymium (Pr) | 0.03 (0.02, 0.03) | 0.03 (0.02, 0.03) | 0.03 (0.02, 0.03) | 0.37 |
| Cobalt (Co) | 0.41 (0.30, 0.53) | 0.33 (0.25, 0.42) | 0.47 (0.36, 0.61) | <0.01 |
| Nickel (Ni) | 3.28 (2.46, 4.49) | 3.14 (2.30, 4.23) | 3.42 (2.60, 4.67) | <0.01 |

**Table S2** Single heavy metal model results

| Heavy metals | Quintiles | | | | |
| --- | --- | --- | --- | --- | --- |
|  | Q1 | Q2 | Q3 | Q4 | Q5 |
| Ag (ng/ml) | ≤ 0.06 | 0.06 ~ 0.09 | 0.09 ~ 0.11 | 0.11 ~ 0.16 | > 0.16 |
| n (controls/cases) | 393 / 13 | 378 / 27 | 388 / 17 | 382 / 23 | 381 / 25 |
| Crude | 1.00 (reference) | 2.16 (1.09 ~ 4.25) | 1.32 (0.63 ~ 2.79) | 1.82 (0.92 ~ 3.62) | 1.97 (0.99 ~ 3.92) |
| Model Ⅰ** | 1.00 (reference) | 2.18 (1.10 ~ 4.30) | 1.34 (0.64 ~ 2.81) | 1.99 (0.99 ~ 4.03) | 2.06(1.03 ~ 4.11) |
| Sn (ng/ml) | ≤ 0.34 | 0.34 ~ 0.5 | 0.5 ~ 0.7 | 0.7 ~ 1 | > 1 |
| n (controls/cases) | 394 / 12 | 380 / 25 | 387 / 18 | 380 / 25 | 381 / 25 |
| Crude | 1.00 (reference) | 2.16 (1.07 ~ 4.37) | 1.52 (0.72 ~ 3.21) | 2.16 (1.07 ~ 4.37) | 2.16 (1.07 ~ 4.37) |
| Model Ⅰ* | 1.00 (reference) | 2.27 (1.12 ~ 4.6) | 1.54 (0.73 ~ 3.24) | 2.18 (1.08 ~ 4.42) | 2.18 (1.08 ~ 4.42) |
| Sb (ng/ml) | ≤ 4.13 | 4.13 ~ 4.54 | 4.54 ~ 5.04 | 5.04 ~ 5.82 | > 5.82 |
| n (controls/cases) | 389 / 17 | 386 / 19 | 383 / 22 | 383 / 22 | 381 / 25 |
| Crude | 1.00 (reference) | 1.13 (0.58 ~ 2.2) | 1.31 (0.69 ~ 2.5) | 1.31 (0.69 ~ 2.5) | 1.51 (0.8 ~ 2.82) |
| Model Ⅰ* | 1.00 (reference) | 1.22 (0.62 ~ 2.43) | 1.31 (0.67 ~ 2.55) | 1.34 (0.7 ~ 2.55) | 1.54 (0.81 ~ 2.94) |
| Nd (ng/ml) | ≤ 0.08 | 0.08 ~ 0.11 | 0.11 ~ 0.13 | 0.13 ~ 0.17 | > 0.17 |
| n (controls/cases) | 386 / 20 | 390 / 15 | 384 / 21 | 386 / 21 | 376 / 28 |
| Crude | 1.00 (reference) | 0.74 (0.37 ~ 1.47) | 1.05 (0.56 ~ 1.97) | 1.05 (0.56 ~ 1.97) | 1.43 (0.8 ~ 2.58) |
| Model Ⅰ* | 1.00 (reference) | 0.73 (0.36 ~ 1.49) | 1.07 (0.56 ~ 2.05) | 1.04 (0.55 ~ 1.99) | 1.52 (0.83 ~ 2.79) |
| Zn (ng/ml) | ≤ 4660.2 | 4660.2 ~ 5286.17 | 5286.17 ~ 5818.92 | 5818.92 ~ 6492.17 | > 6492.17 |
| n (controls/cases) | 381 / 25 | 383 / 22 | 386 / 19 | 382 / 23 | 390 / 16 |
| Crude | 1.00 (reference) | 0.88 (0.49 ~ 1.58) | 0.75 (0.41 ~ 1.37) | 0.91 (0.51 ~ 1.65) | 0.63 (0.33 ~ 1.19) |
| Model Ⅰ* | 1.00 (reference) | 0.85 (0.46 ~ 1.56) | 0.72 (0.38 ~ 1.35) | 0.84 (0.46 ~ 1.55) | 0.58 (0.3 ~ 1.13) |
| Fe (ng/ml) | ≤ 415718.97 | 415718.97 ~ 477067.84 | 477067.84 ~ 523143.7 | 523143.7 ~ 574079.6 | > 574079.6 |
| n (controls/cases) | 383 / 23 | 382 / 23 | 385 / 20 | 386 / 19 | 386 / 20 |
| Crude | 1.00 (reference) | 1 (0.56 ~ 1.8) | 0.87 (0.47 ~ 1.6) | 0.82 (0.44 ~ 1.53) | 0.86 (0.47 ~ 1.58) |
| Model Ⅰ* | 1.00 (reference) | 1 (0.54 ~ 1.84) | 0.79 (0.42 ~ 1.52) | 0.78 (0.39 ~ 1.55) | 0.79 (0.4 ~ 1.56) |
| Cr (ng/ml) | ≤ 0.97 | 0.97 ~ 1.68 | 1.68 ~ 2.6 | 2.6 ~ 4.3 | > 4.3 |
| n (controls/cases) | 387 / 19 | 381 / 24 | 381 / 24 | 384 / 21 | 389 / 17 |
| Crude | 1.00 (reference) | 1.28 (0.69 ~ 2.4) | 1.28 (0.69 ~ 2.4) | 1.12 (0.6 ~ 2.09) | 0.89 (0.46 ~ 1.73) |
| Model Ⅰ* | 1.00 (reference) | 1.34 (0.71 ~ 2.5) | 1.26 (0.67 ~ 2.36) | 1.09 (0.57 ~ 2.09) | 0.85 (0.43 ~ 1.69) |
| Ge (ng/ml) | ≤ 55.39 | 55.39 ~ 68.94 | 68.94 ~ 80.23 | 80.23 ~ 96.99 | > 96.99 |
| n (controls/cases) | 384 / 22 | 387 / 18 | 386 / 19 | 387 / 18 | 378 / 28 |
| Crude | 1.00 (reference) | 0.81 (0.42 ~ 1.55) | 0.86 (0.46 ~ 1.61) | 0.81 (0.42 ~ 1.55) | 1.3 (0.73 ~ 2.29) |
| Model Ⅰ* | 1.00 (reference) | 0.8 (0.42 ~ 1.53) | 0.89 (0.46 ~ 1.69) | 0.82 (0.42 ~ 1.59) | 1.3 (0.72 ~ 2.33) |
| Mo (ng/ml) | ≤ 1.11 | 1.11 ~ 1.35 | 1.35 ~ 1.6 | 1.6 ~ 1.95 | > 1.95 |
| n (controls/cases) | 384 / 22 | 384 / 21 | 385 / 20 | 378 / 27 | 391 / 15 |
| Crude | 1.00 (reference) | 0.95 (0.52 ~ 1.75) | 0.9 (0.48 ~ 1.69) | 1.25 (0.69 ~ 2.24) | 0.67 (0.34 ~ 1.31) |
| Model Ⅰ* | 1.00 (reference) | 0.86 (0.46 ~ 1.61) | 0.79 (0.42 ~ 1.49) | 1.13 (0.63 ~ 2.03) | 0.64 (0.32 ~ 1.27) |
| Cd (ng/ml) | ≤ 0.98 | 0.98 ~ 1.18 | 1.18 ~ 1.36 | 1.36 ~ 1.62 | > 1.62 |
| n (controls/cases) | 385 / 21 | 388 / 17 | 388 / 17 | 375 / 30 | 386 / 20 |
| Crude | 1.00 (reference) | 0.8 (0.42 ~ 1.53) | 0.8 (0.42 ~ 1.53) | 1.46 (0.83 ~ 2.58) | 0.95 (0.51 ~ 1.78) |
| Model Ⅰ* | 1.00 (reference) | 0.81 (0.42 ~ 1.58) | 0.79 (0.4 ~ 1.53) | 1.58 (0.88 ~ 2.85) | 0.82 (0.43 ~ 1.56) |
| Cu (ng/ml) | ≤ 749.75 | 749.75 ~ 820 | 820 ~ 889.08 | 889.08 ~ 976.91 | > 976.91 |
| n (controls/cases) | 386 / 20 | 379 / 26 | 386 / 19 | 383 / 22 | 388 / 18 |
| Crude | 1.00 (reference) | 1.32 (0.72 ~ 2.43) | 0.95 (0.5 ~ 1.82) | 1.11 (0.59 ~ 2.07) | 0.9 (0.47 ~ 1.71) |
| Model Ⅰ* | 1.00 (reference) | 1.27 (0.69 ~ 2.33) | 0.9 (0.47 ~ 1.73) | 1.07 (0.56 ~ 2.05) | 0.84 (0.42 ~ 1.66) |
| Hg (ng/ml) | ≤ 0.49 | 0.49 ~ 0.66 | 0.66 ~ 0.82 | 0.82 ~ 1.07 | > 1.07 |
| n (controls/cases) | 385 / 21 | 385 / 20 | 384 / 21 | 386 / 19 | 382 / 24 |
| Crude | 1.00 (reference) | 0.95 (0.51 ~ 1.78) | 1 (0.53 ~ 1.87) | 0.9 (0.48 ~ 1.69) | 1.15 (0.63 ~ 2.11) |
| Model Ⅰ* | 1.00 (reference) | 0.9 (0.48 ~ 1.68) | 1.05 (0.56 ~ 1.97) | 0.89 (0.46 ~ 1.69) | 1.28 (0.69 ~ 2.4) |
| Mn (ng/ml) | ≤ 12.11 | 12.11 ~ 14.46 | 14.46 ~ 16.99 | 16.99 ~ 20.42 | > 20.42 |
| n (controls/cases) | 391 / 15 | 376 / 29 | 378 / 27 | 388 / 17 | 389 / 17 |
| Crude | 1.00 (reference) | 2.01 (1.05 ~ 3.85) | 1.86 (0.97 ~ 3.55) | 1.14 (0.56 ~ 2.31) | 1.14 (0.56 ~ 2.31) |
| Model Ⅰ* | 1.00 (reference) | 2.2 (1.13 ~ 4.29) | 2.14 (1.1 ~ 4.16) | 1.12 (0.54 ~ 2.31) | 1.13 (0.54 ~ 2.37) |
| La (ng/ml) | ≤ 0.02 | 0.02 ~ 0.03 | 0.03 ~ 0.04 | 0.04 ~ 0.06 | > 0.06 |
| n (controls/cases) | 390 / 16 | 385 / 20 | 374 / 31 | 387 / 18 | 386 / 20 |
| Crude | 1.00 (reference) | 1.27 (0.65 ~ 2.48) | 2.01 (1.08 ~ 3.77) | 1.14 (0.57 ~ 2.26) | 1.26 (0.65 ~ 2.45) |
| Model Ⅰ* | 1.00 (reference) | 1.26 (0.63 ~ 2.5) | 1.97 (1.05 ~ 3.7) | 1.19 (0.59 ~ 2.4) | 1.38 (0.69 ~ 2.73) |
| Ce (ng/ml) | ≤ 0.08 | 0.08 ~ 0.1 | 0.1 ~ 0.12 | 0.12 ~ 0.16 | > 0.16 |
| n (controls/cases) | 391 / 15 | 389 / 16 | 375 / 30 | 374 / 31 | 393 / 13 |
| Crude | 1.00 (reference) | 1.07 (0.52 ~ 2.21) | 2.08 (1.11 ~ 3.89) | 2.16 (1.15 ~ 4.04) | 0.86 (0.4 ~ 1.85) |
| Model Ⅰ* | 1.00 (reference) | 1.12 (0.54 ~ 2.31) | 2.08 (1.09 ~ 3.96) | 2.32 (1.21 ~ 4.42) | 0.9 (0.42 ~ 1.94) |
| Pr (ng/ml) | ≤ 0.02 | 0.02 ~ 0.02 | 0.02 ~ 0.03 | 0.03 ~ 0.04 | > 0.04 |
| n (controls/cases) | 386 / 21 | 384 / 20 | 391 / 15 | 382 / 23 | 379 / 26 |
| Crude | 1.00 (reference) | 0.96 (0.51 ~ 1.8) | 0.7 (0.35 ~ 1.4) | 1.11 (0.6 ~ 2.03) | 1.26 (0.7 ~ 2.27) |
| Model Ⅰ* | 1.00 (reference) | 0.95 (0.51 ~ 1.78) | 0.68 (0.34 ~ 1.36) | 1.13 (0.6 ~ 2.11) | 1.3 (0.71 ~ 2.38) |
| Co (ng/ml) | ≤ 0.28 | 0.28 ~ 0.37 | 0.37 ~ 0.45 | 0.45 ~ 0.57 | > 0.57 |
| n (controls/cases) | 381 / 25 | 389 / 16 | 380 / 25 | 383 / 22 | 389 / 17 |
| Crude | 1.00 (reference) | 0.63 (0.33 ~ 1.19) | 1 (0.57 ~ 1.77) | 0.88 (0.49 ~ 1.58) | 0.66 (0.35 ~ 1.24) |
| Model Ⅰ* | 1.00 (reference) | 0.64 (0.33 ~ 1.22) | 1.03 (0.57 ~ 1.86) | 0.88 (0.47 ~ 1.64) | 0.64 (0.32 ~ 1.27) |
| Ni (ng/ml) | ≤ 2.28 | 2.28 ~ 2.97 | 2.97 ~ 3.64 | 3.64 ~ 4.9 | > 4.9 |
| n (controls/cases) | 388 / 18 | 383 / 22 | 386 / 19 | 379 / 26 | 386 / 20 |
| Crude | 1.00 (reference) | 1.23 (0.65 ~ 2.36) | 1.06 (0.55 ~ 2.07) | 1.48 (0.8 ~ 2.71) | 1.12 (0.58 ~ 2.13) |
| Model Ⅰ* | 1.00 (reference) | 1.22 (0.64 ~ 2.33) | 1.13 (0.58 ~ 2.2) | 1.54 (0.82 ~ 2.88) | 1.09 (0.56 ~ 2.13) |

*, The logistic model was adjusted for age, sex, BMI, smoking, alcohol consumption, physical activity, family incom

**Table S3** Qgcomp Model: Association between heavy metal mixtures and depressive symptoms.

| Model* | Estimate | Std. Error | OR | 95%CI |
| --- | --- | --- | --- | --- |
| Ag + Sb + Sn + Ce + La + Sn^2 + Ce^2 + La*Ce | 0.308 | 0.130 | 1.361 | (1.106, 1.615) |

*, The qgcomp model was adjusted for age, sex, BMI, smoking, alcohol consumption, physical activity, family income.


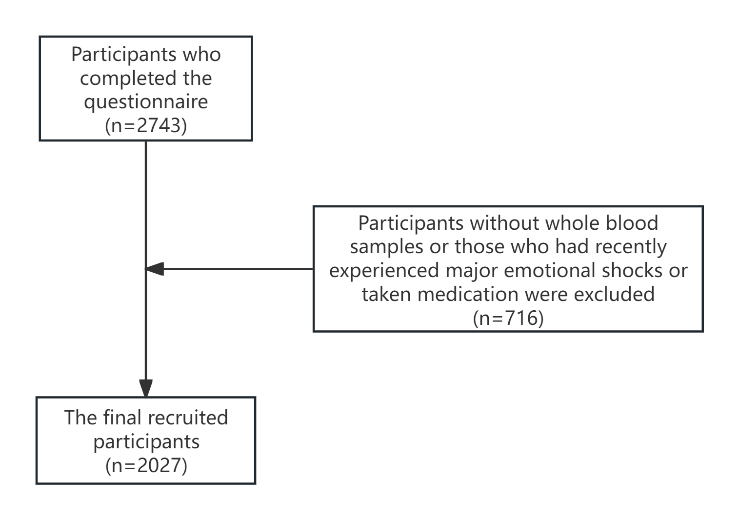


**Figure S1** Sample selection flow chart


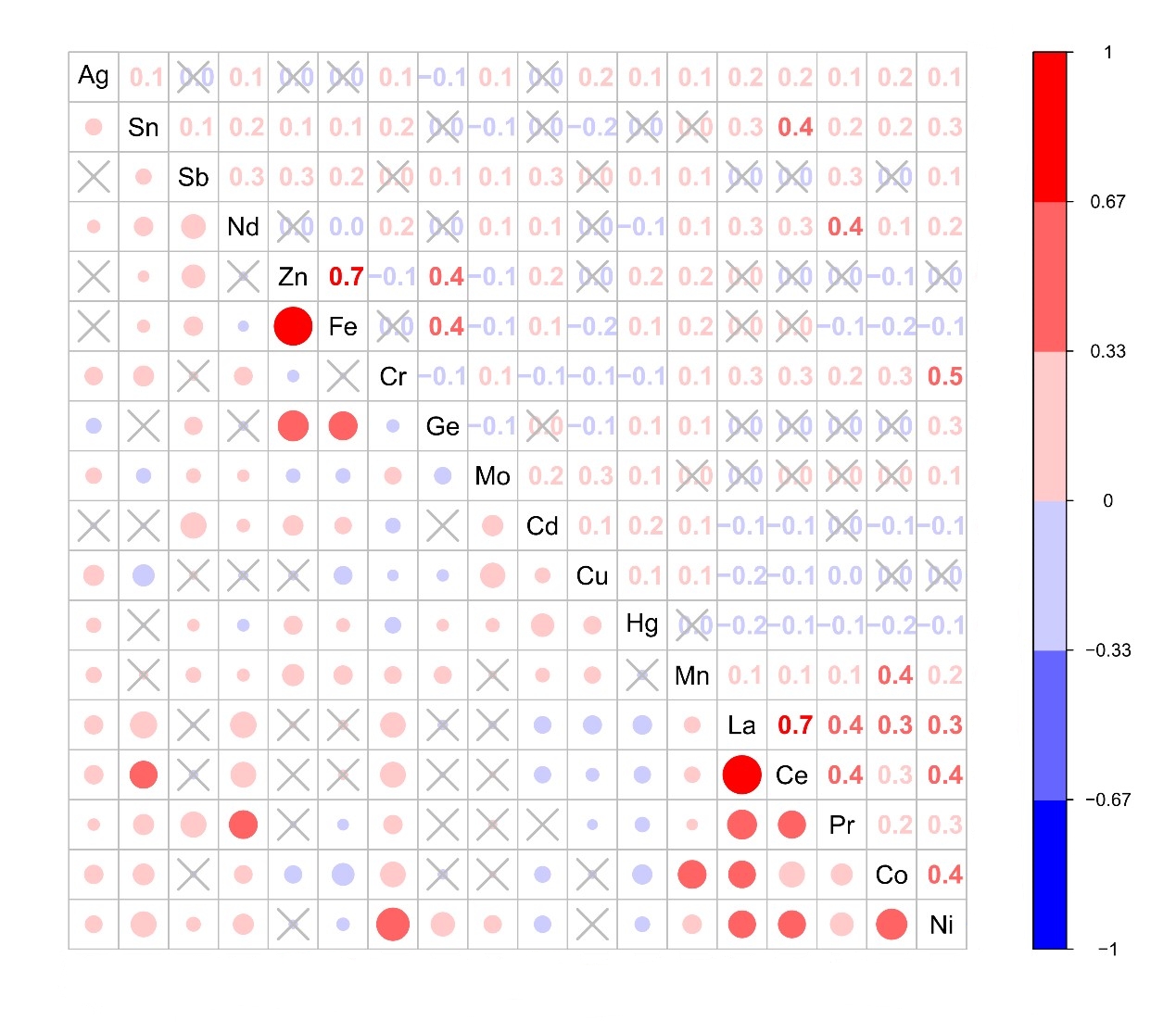


**Figure S2** The correlation among heavy metals. Number, Spearman correlation coefficient; dots, Spearman's correlation coefficient values; blue, negative value of Spearman's correlation coefficient; red, positive value of Spearman's correlation coefficient. ×, *p*-value corresponding to correlation coefficient is greater than 0.05.


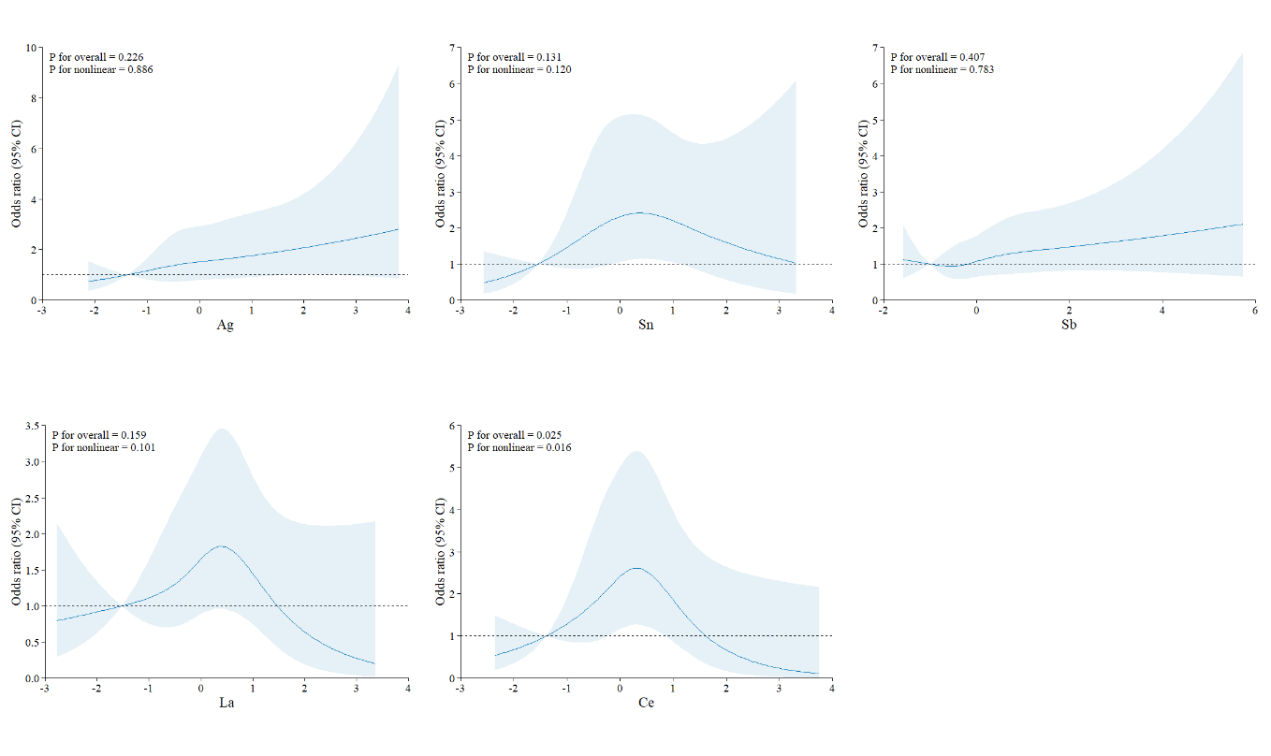


**Figure S****3** Cubic spline modeling of five depressive symptom-related metals (Ag, Sb, Sn, La, and Ce). Ag and Sb showed a positive correlation with depressive symptoms, a nonlinear relationship between Ce and depressive symptoms, and an approximate nonlinear relationship between Sn or La and depressive symptoms.


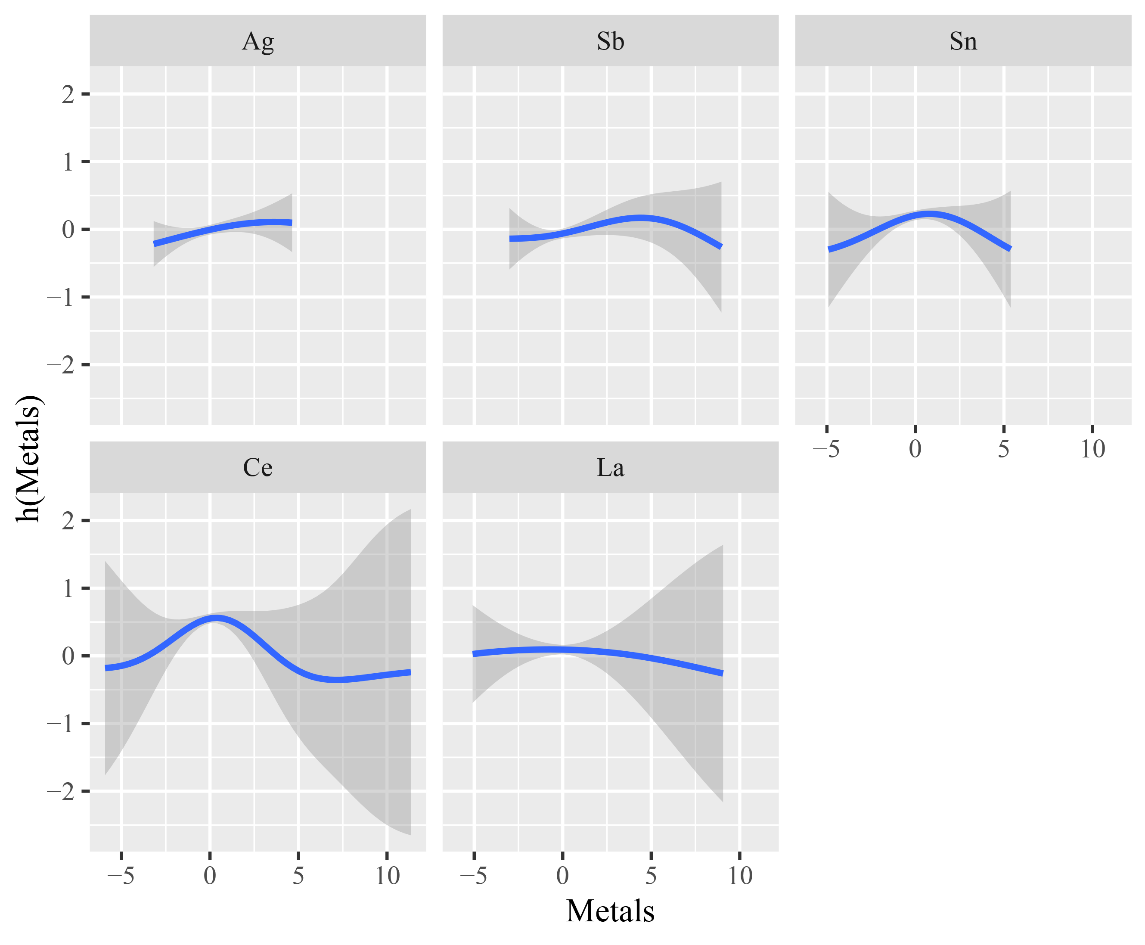


**Figure S4.** BKMR model to assess nonlinear associations of five heavy metal exposure levels with depressive symptoms.


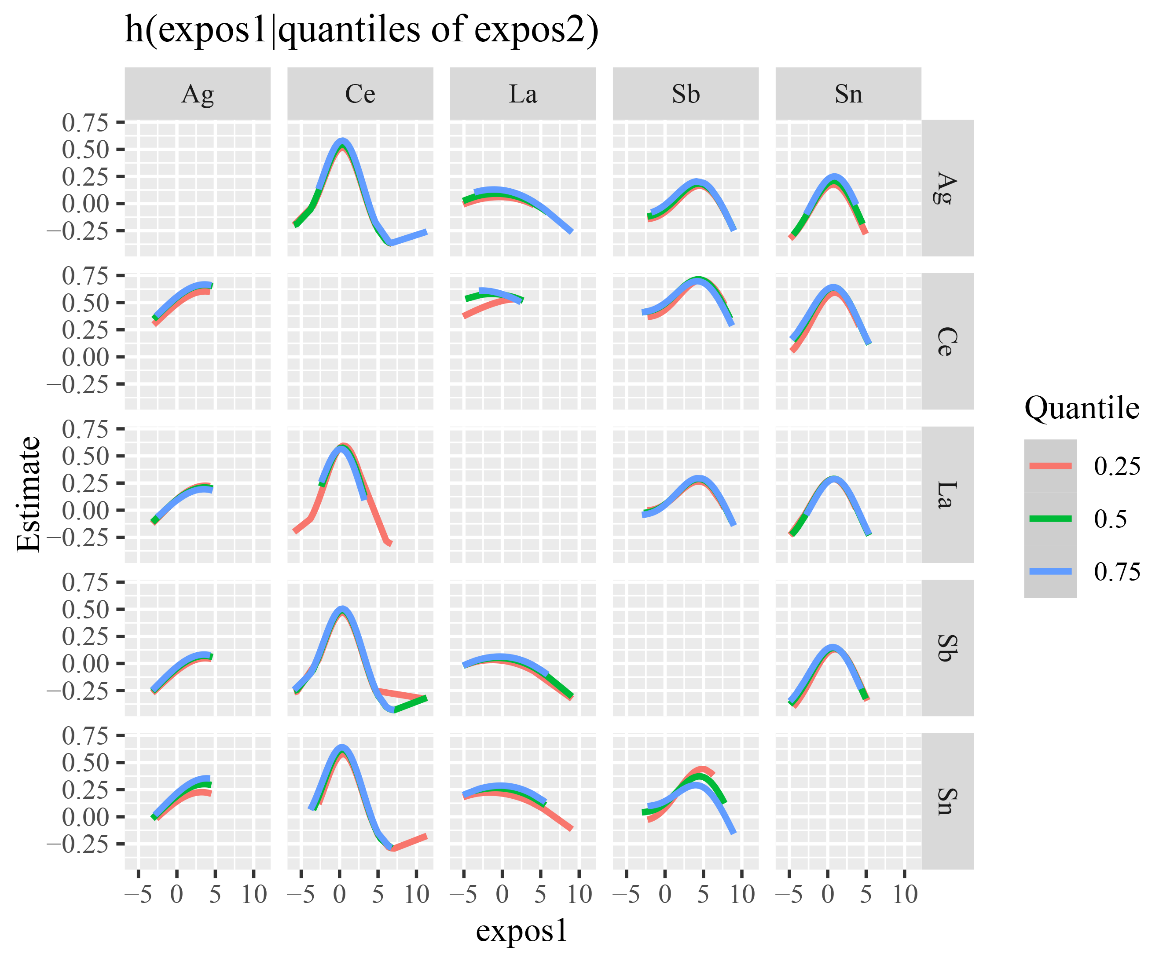


**Figure S5** Results of interaction between heavy metals. When Ce was at the 25th percentile, increased La exposure increased the incidence of depressive symptoms, when Ce was at the 50th percentile, the effect of increased La exposure on depressive symptoms tended to be flat, and when Ce was at the 75th percentile, increased La exposure decreased the incidence of depressive symptoms.

**
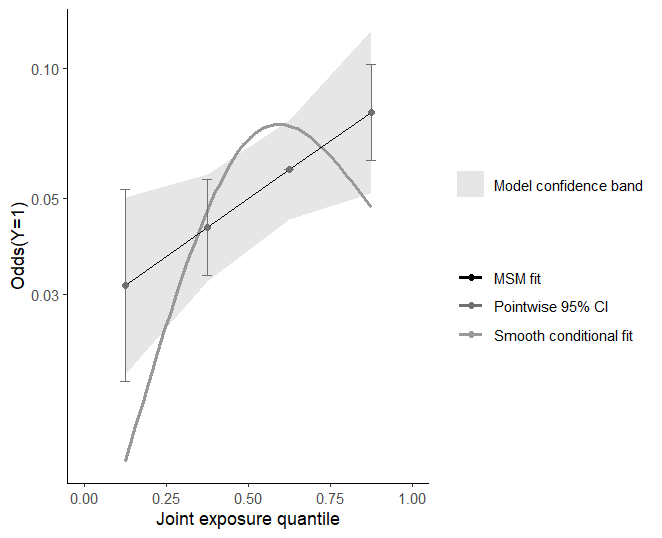
**

**Figure S6.** The Qgcomp method assesses the association between mixed exposure to five metals and depressive symptoms. (A total of eight predictors were included, including the main effects of five metals, two quadratic terms, and one interaction term).


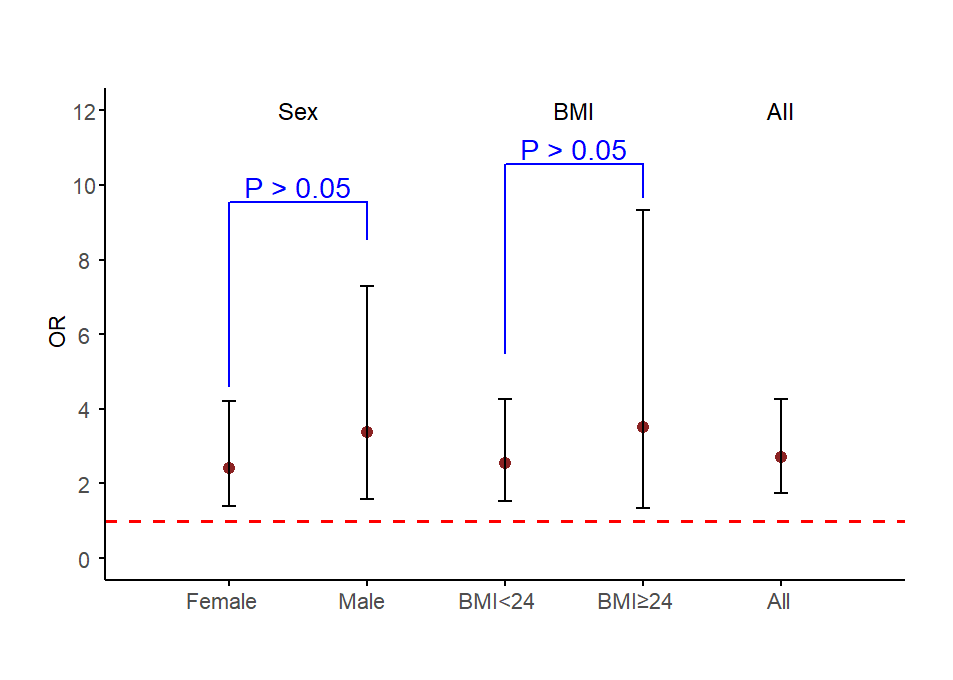


**Figure S7** Relationship between ERS and depressive symptoms in different subgroups.


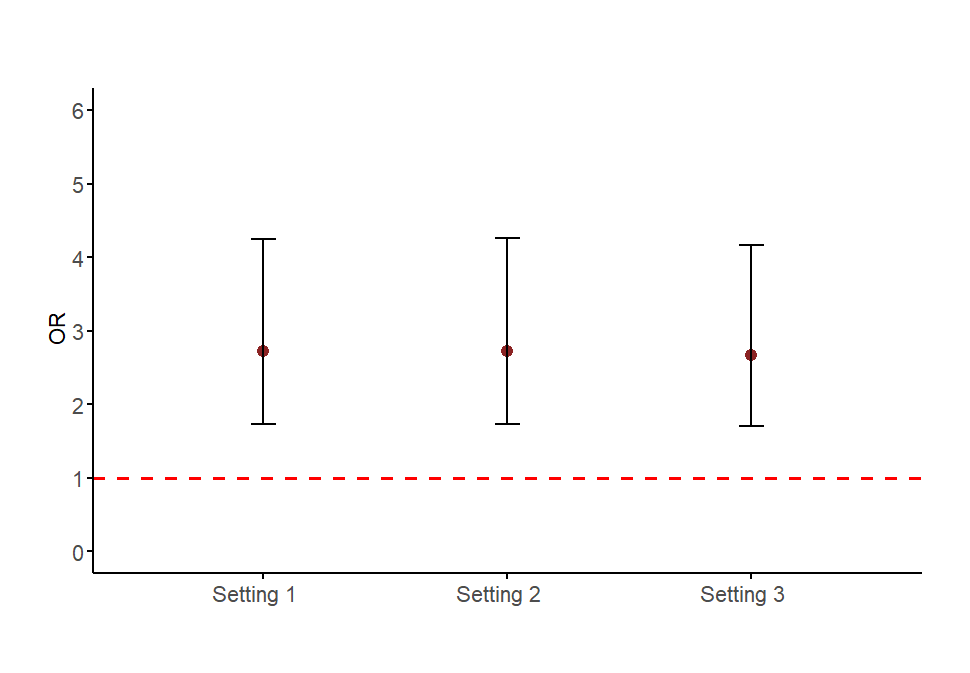


**Figure S8** Sensitivity analysis of ERS and risk of depression. Setting 1, unadjusted model; Setting 2, main model adjusted for sex, age, BMI, exercise, presence of tobacco, presence of alcohol, household income. Setting 3, history of heart disease, hyperlipidemia, hypertension, diabetes included in the main model.


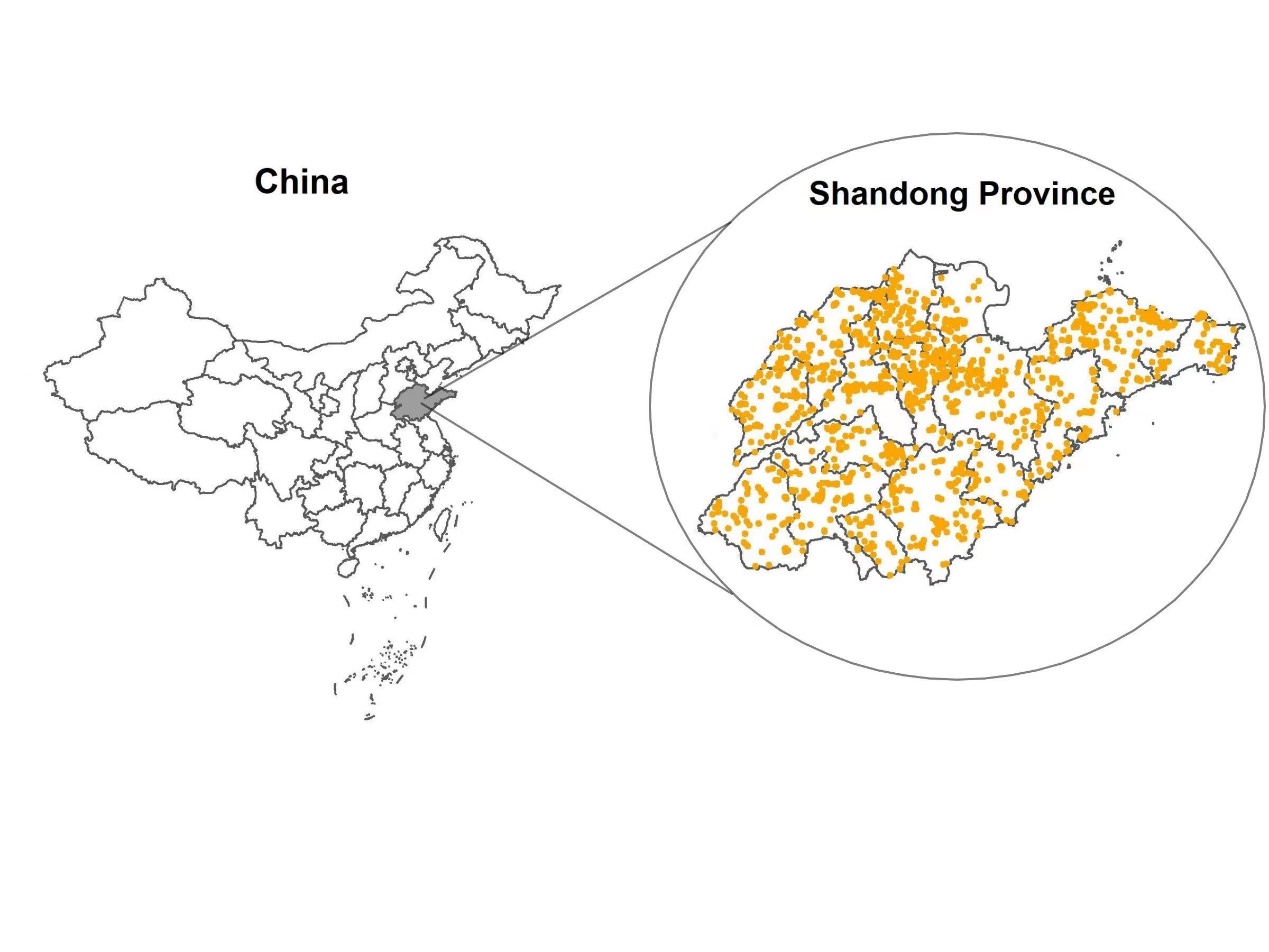


**Figure S9.** Distribution of study subjects across Shandong Province.

**Elemental detection**

On the scheduled morning, 10 ml of fasting peripheral venous blood was collected from each participant at the university hospital and stored at -80°C for backup. An inductively coupled plasma-mass spectrometer (ICP-MS, ELAN DRC II, PerkinElmer, USA) was used to measure the levels of the elements. In brief, blood samples (0.35 ml) were transferred into quartz tubes and then mixed with 0.40 ml of nitric acid. We pre-digested the quartz tubes at ambient temperature for two hours and then placed them in an Ultra WAVE microwave digestion system (Ultra WAVE, Milestone Co., Italy) for 50 minutes. Afterward, we added 0.1 ml of internal standard elements (Indium: 2 ng/ml) and diluted to 8 ml with ultrapure water. The analysis was performed in the Central Laboratory of Biological Elements in the Peking University Health Science Center certified by the Chinese Metrology Accreditation (CMA) system.

Metal quality control measures were as follows: Reagent blanks and procedure blanks were prepared for each analytical batch to eliminate potential background contamination. The limit of detection (LOD) for metals was calculated as 3 times the standard deviation (SD) of 11 replicate determinations of the blank sample. During the experiment, 6 national standard materials of quality control were simultaneously determined. All the measured values of all the elements were in line with the identification values (or reference values) of the national standard materials. The recoveries of elements were between 95% and 105%, and the precision of element determination was less than 5%, indicating that the determination method in this study was accurate and reliable.

The limits of detection of Ag, Sb, Sn, La and Ce were 0.0020, 0.0030, 0.0070, 0.0003 and 0.0004 µg/L. The limits of quantitation of Ag, Sb, Sn, La and Ce were 0.0067 , 0.0100 , 0.0233, 0.0010 and 0.0013 µg/ L.
